# Editorial: Adaptive eating behaviors and energy intake: coping strategies and their impact on health and performance

**DOI:** 10.3389/fspor.2025.1746671

**Published:** 2026-01-12

**Authors:** Caio Eduardo Gonçalves Reis, Ragami Chaves Alves, Wilson Max Almeida Monteiro de Moraes

**Affiliations:** 1 Lifestyle Nutrition Research Group, Department of Nutrition, Universidade de Brasília, Brasília, Brazil; 2 Department of Physical Education, Metabolism, Nutrition and Strength Training Research Group, Federal University of Paraná, Curitiba, Brazil; 3 Graduate Program in Physical Education, Catholic University of Brasília, Brasília, Brazil

**Keywords:** disordered eating behavior, energy availability, sports nutrition, relative energy deficiency in sport, energy intake, athletic performance

Adaptive eating behaviors represent an interesting and critical research topic in nutrition and sports science. Athletes and physically active individuals need to balance energy intake and expenditure (diet and supplement intake with training load) to improve performance and recovery. The ability to adjust eating behavior in response to physiological and psychological demands presented an important challenge for athletes (1). Historically, the literature has focused on maladaptive responses, such as restrictive eating or disordered patterns, while neglecting to explore the adaptive spectrum of coping and regulation (2).

**Figure 1:**
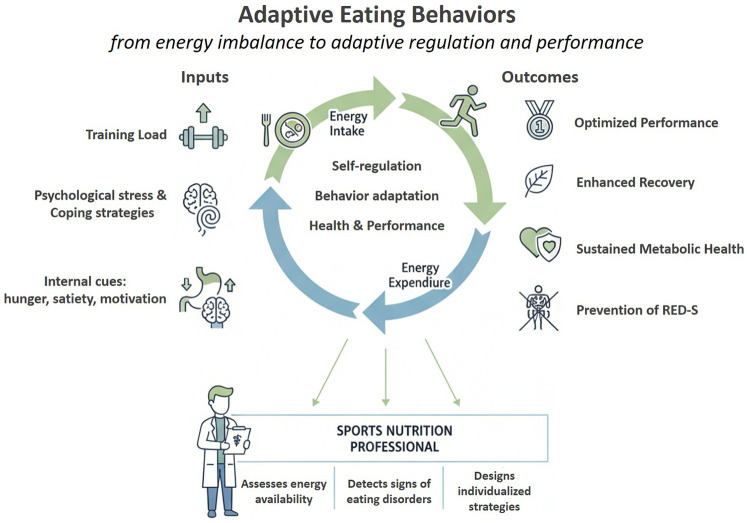
Conceptual model of adaptive eating behaviors in sports and performance. Adaptive regulation of energy intake and expenditure results from the interaction between internal cues (e.g., hunger, satiety, motivation), psychological coping strategies, and training load. These factors drive behavioral flexibility and self-regulation, supporting performance, recovery, and long-term metabolic health. The sports nutrition professional plays a central role in this process—assessing energy availability, detecting early signs of disordered eating, and designing individualized fueling strategies to prevent RED-S and optimize performance.

Therefore, the Research Topic “Adaptive Eating Behaviors and Energy Intake – Coping Strategies and Their Impact on Health and Performance” was proposed to address the evidence gap in the field, emphasizing the importance of understanding how individuals manage their energy intake under high training loads and competitive schedules. The main objective was to provide a comprehensive perspective of adaptive eating behaviors and energy intake, encompassing both physiological and behavioral aspects, to highlight their relevance in clinical nutrition and sports performance (Figure 1).

In this context, the article by Wachten et al. provided important insights for the topic, examining the interplay between orthorexia nervosa, exercise addiction, and well-being across different sports and genders (*n* = 1,064). The authors highlight that excessive health behaviors, while sometimes aimed at optimizing performance and health, can negatively impact psychological well-being, particularly among female athletes (Wachten et al.). They presented a nuanced perspective on how maladaptive eating and exercise patterns influence athletes' holistic health. Preventive measures should consider gender-specific risks in excessive health behaviors across fitness, resistance, and endurance sports.

Additionally, Xie et al. examined the effects of varying resistance training intensities on body composition and nutritional intake, highlighting how different training strategies influence body composition under energy deficit conditions. Their findings underscore that while combined exercise approaches are most effective for preserving lean mass and reducing fat mass, careful attention to energy intake is crucial, as caloric restriction can predispose athletes to low energy availability (Xie et al.). These results reinforce the importance of adaptive eating behaviors to align energy intake with training demands and support both performance and metabolic health.

The resistance training viewpoint, Wang et al. conducted a cluster-randomized controlled trial to examine the effects of varying resistance training intensities on body composition and nutritional intake among overweight and obese college women. The authors highlight that high-intensity resistance training not only promotes the greatest improvements in lean mass and fat loss but also interacts with energy intake patterns, emphasizing the need to align exercise with dietary strategies to prevent negative adaptive eating behaviors (Wang et al.). They provide a perspective on how adaptive eating behaviors, aligned with tailored exercise interventions, can support metabolic health, optimize body composition, and ensure energy intake meets training demands to enhance both performance and well-being.

Finally, Zdzieblik et al. conducted a pilot trial examining the effects of a gluten-free diet on performance outcomes in non-celiac athletes undergoing sprint interval training. Their findings indicate that a gluten-free diet *per si* does not affect performance outcomes, suggesting that such dietary interventions may not be necessary for enhancing performance in this population (Zdzieblik et al.). This result stresses the importance of evidence-based dietary strategies to improve athletic performance and highlights the need for individualized nutrition interventions that align with athletes' specific needs and goals.

When taken collectively, these contributions underscore a paradigm shift in sports nutrition: moving from a predominant focus on energy deficiency and maladaptive behaviors toward understanding adaptive regulation as a continuum that optimizes performance and health. They emphasize that adaptive eating is not static, but dynamically shaped by the interaction of training load, recovery, motivation, and internal cues of hunger and satiety. This concept is gaining traction in the scientific literature. For instance, de Moraes et al. (2024) investigated adaptive responses in energy intake regulation using a refeed model among competitive bodybuilders, highlighting how planned fluctuations in dietary intake can serve as coping strategies to mitigate the physiological and psychological stress of prolonged caloric restriction (3). Their findings illustrate that adaptive eating is not only a compensatory reaction to energy deficits but also a deliberate strategic behavior aimed at restoring energy balance, supporting metabolic flexibility, and preserving performance during intensive training phases.

The findings highlight that adaptive eating in athletes and physically active individuals is not solely a response to physiological cues but also reflects the ability to cope with psychological demands, manage self-perception, and adjust energy intake according to training load and recovery needs. This evidence reinforces the importance of integrating behavioral science, sports psychology, and nutrition to better understand how individuals intrinsically regulate energy intake to support performance and long-term metabolic health.

In sum, the evidence indicates that adaptive eating behaviors are not merely reactive or compensatory; they represent trainable, measurable strategies that allow athletes to sustain performance and long-term health. This Research Topic, therefore, encourages a shift in both research and applied practice – from merely identifying dysfunction to positive adaptive behaviors. Importantly, these findings highlight the crucial role of the sports nutrition professional, who is qualified to assess energy availability, identify early signs of eating disorders, and design an individualized dietary plan to optimize performance and overall health in athletes (4).

As the field advances, future investigations should explore the thresholds of adaptation, identify biomarkers of adaptive regulation, and develop behavioral strategies that accommodate individual variability in energy availability. Bridging mechanistic insights with applied interventions will be essential to fully operationalize adaptive eating as a scientific and practical framework in contemporary sports nutrition.
